# Supplementary material for: Inhibition of FGFR Signaling by Targeting FGF/FGFR Extracellular Interactions: Towards the Comprehension of the Molecular Mechanism through NMR Approaches
Source: Int J Mol Sci. 2022 Sep 17;23(18):10860. doi: 10.3390/ijms231810860 (PMC9503799; doi:10.3390/ijms231810860)
Supplement: Supplementary file 1 [file ijms-23-10860-s001.zip › ijms-1873547-supplementary.pdf]

International Journal of *Molecular Sciences*

MDPI

Supplementary Material

Inhibition of FGFR signaling by targeting FGF/FGFR extracellular interactions: towards the comprehension of the molecular mechanism through NMR approaches

Katiuscia Pagano [1,*], Elisa Longhi [2], Henriette Molinari [1], Giulia Taraboletti [2] and Laura Ragona [1,*]

[1] Istituto di Scienze e Tecnologie Chimiche "Giulio Natta" (SCITEC), via Corti 12, Milano, Italy; laura.ragona@scitec.cnr.it
[2] Laboratory of Tumour Microenvironment, Department of Oncology, Istituto di Ricerche Farmacologiche Mario Negri IRCCS, Bergamo, Italy;
* Correspondence: L.R. laura.ragona@scitec.cnr.it; K.P. katiuscia.pagano@scitec.cnr.it

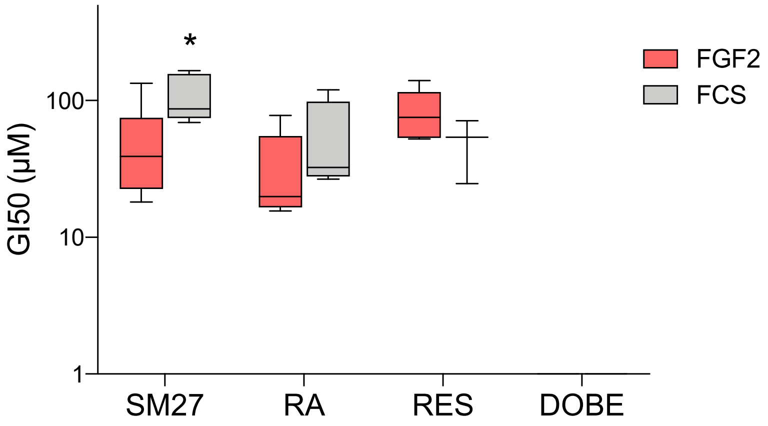

Figure S1. Comparison of the activity of the compounds on EC proliferation induced by FGF2 or FCS. Data are GI50 values (μM). * $p<0.05$ (two-way ANOVA and Tukey's multiple comparison test). Data are from 3-6 independent experiments.

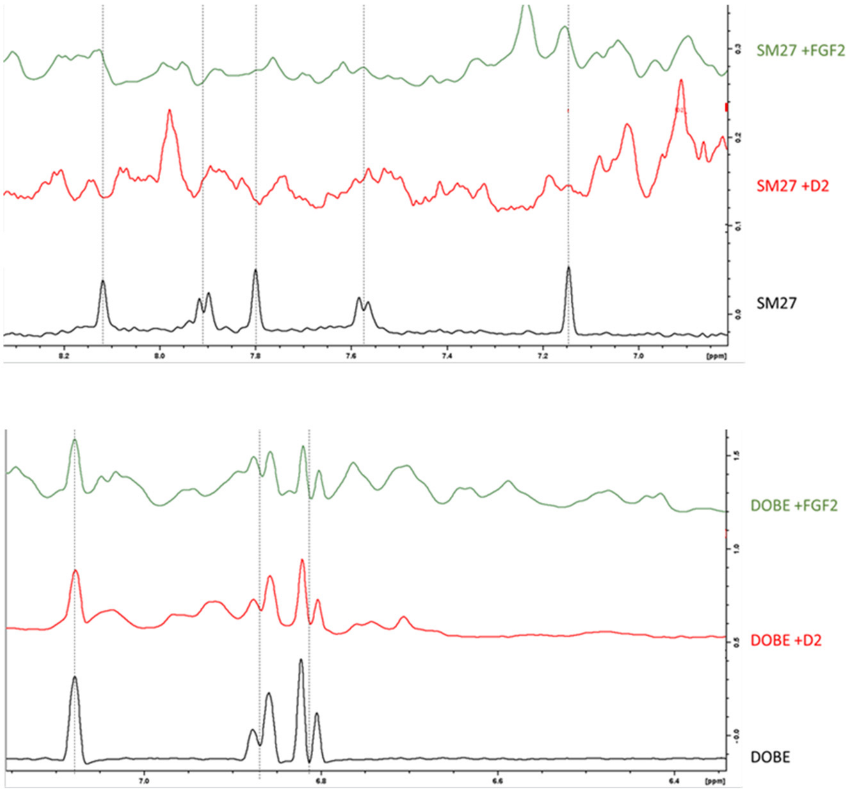

Figure S2. Comparison of 1D 1H spectra of SM27 (upper panel) and DOBE (lower panel), apo (black), and in the presence of D2 (red) or FGF2 (green).

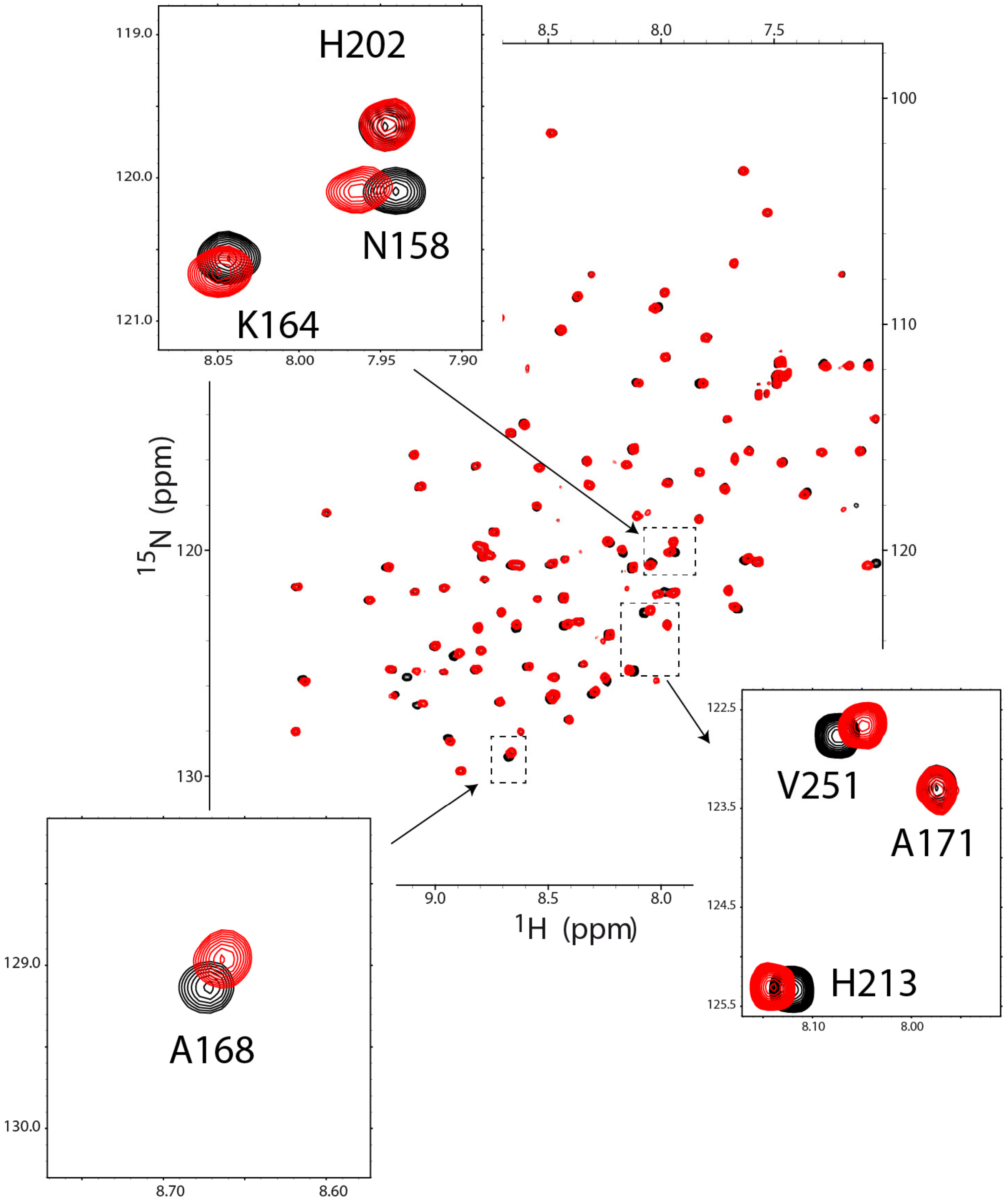

Figure S3. Superimposed ^{1}H-^{15}N HSQC spectra of free 0.2 mM ^{15}N-D2 (black), D2:RA in stoichiometric ratios 1:4 (red). Representative spectral regions are zoomed.

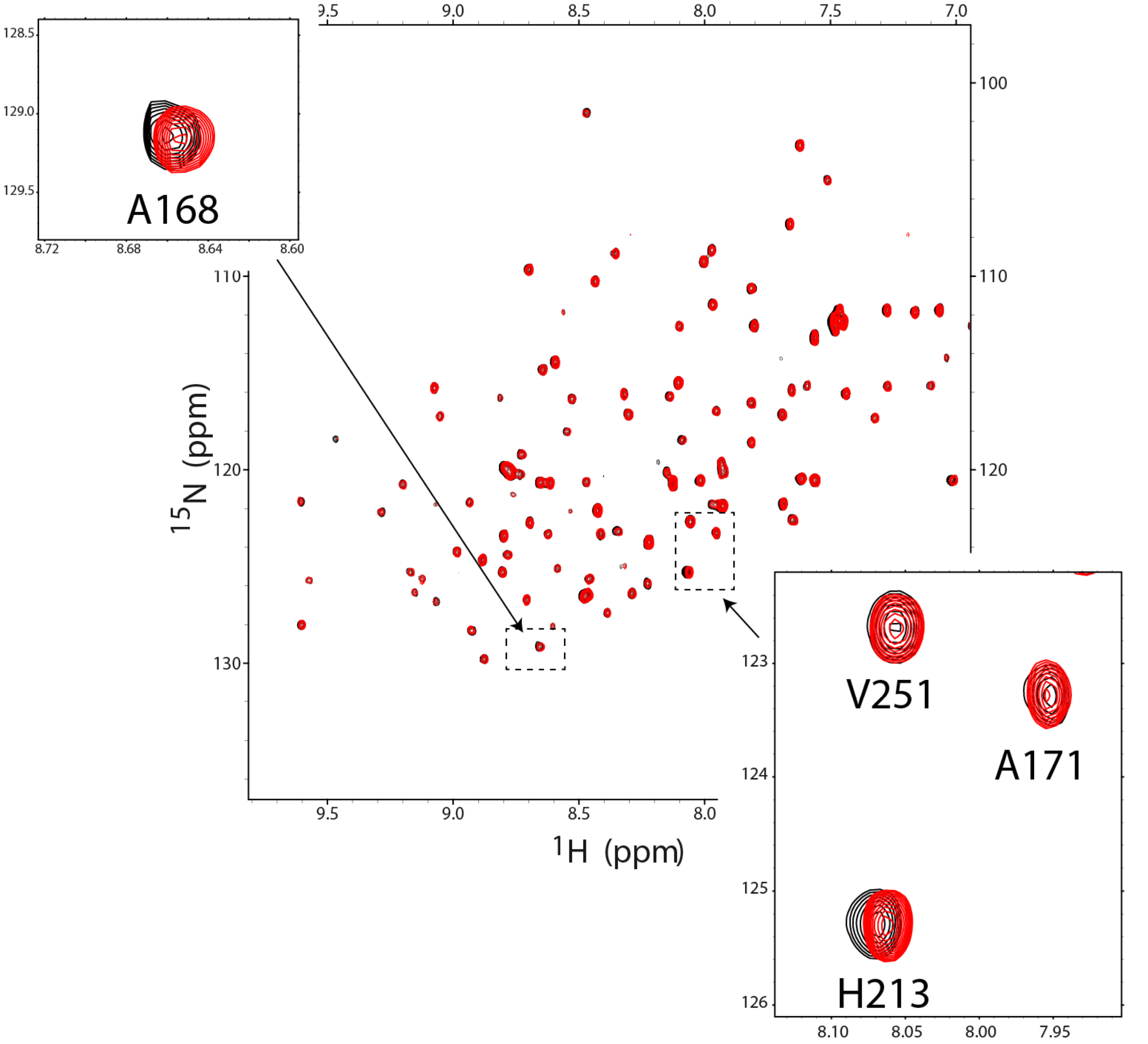

Figure S4. Superimposed ^{1}H-^{15}N HSQC spectra of free 0.2 mM ^{15}N-D2 (black), D2:RES in stoichiometric ratios 1:4 (red). Representative spectral regions are zoomed.

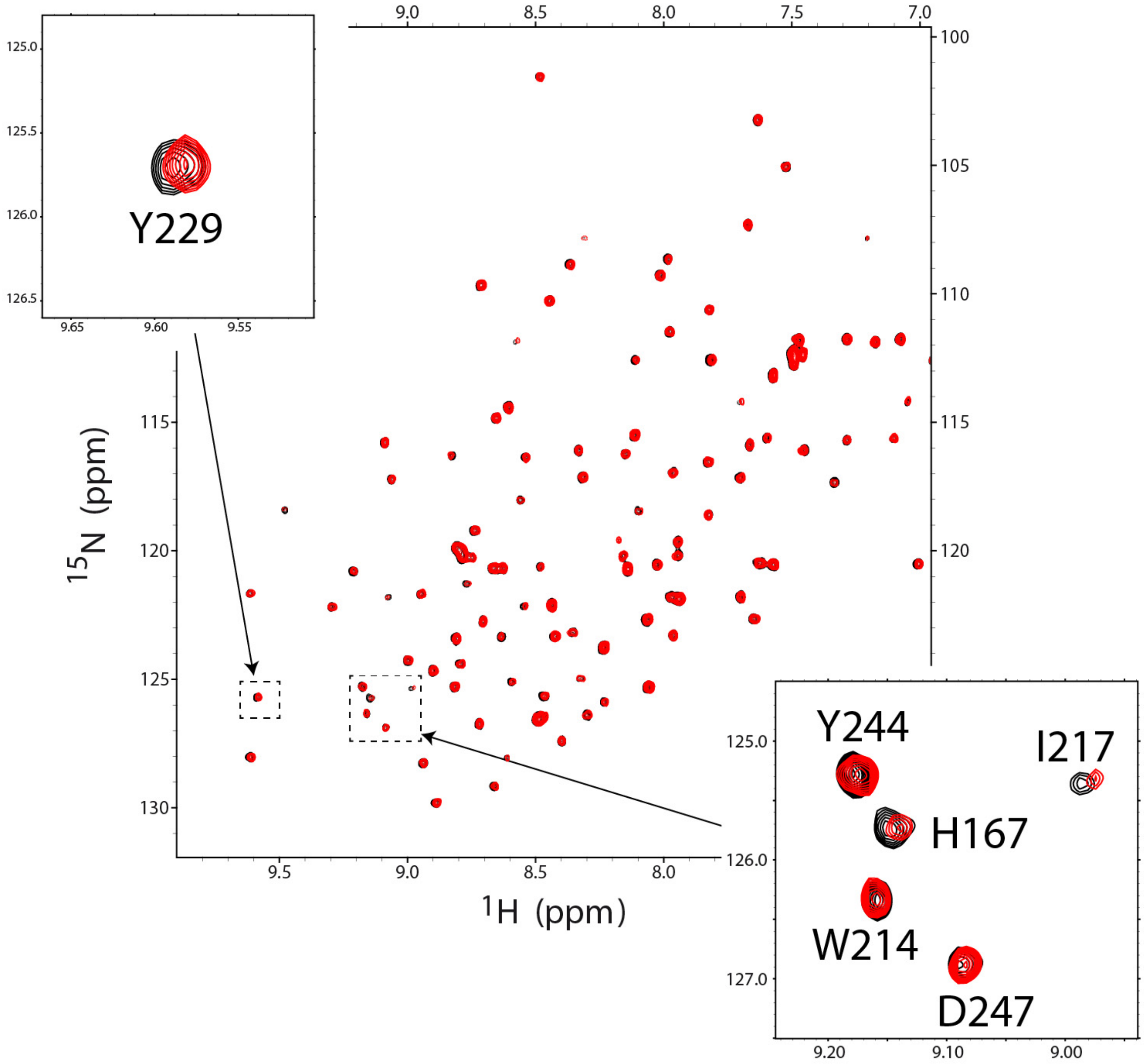

Figure S5. Superimposed ^{1}H-^{15}N HSQC spectra of free 0.2 mM ^{15}N-D2 (black), D2:SM27 in stoichiometric ratios 1:4 (red). Representative spectral regions are zoomed.

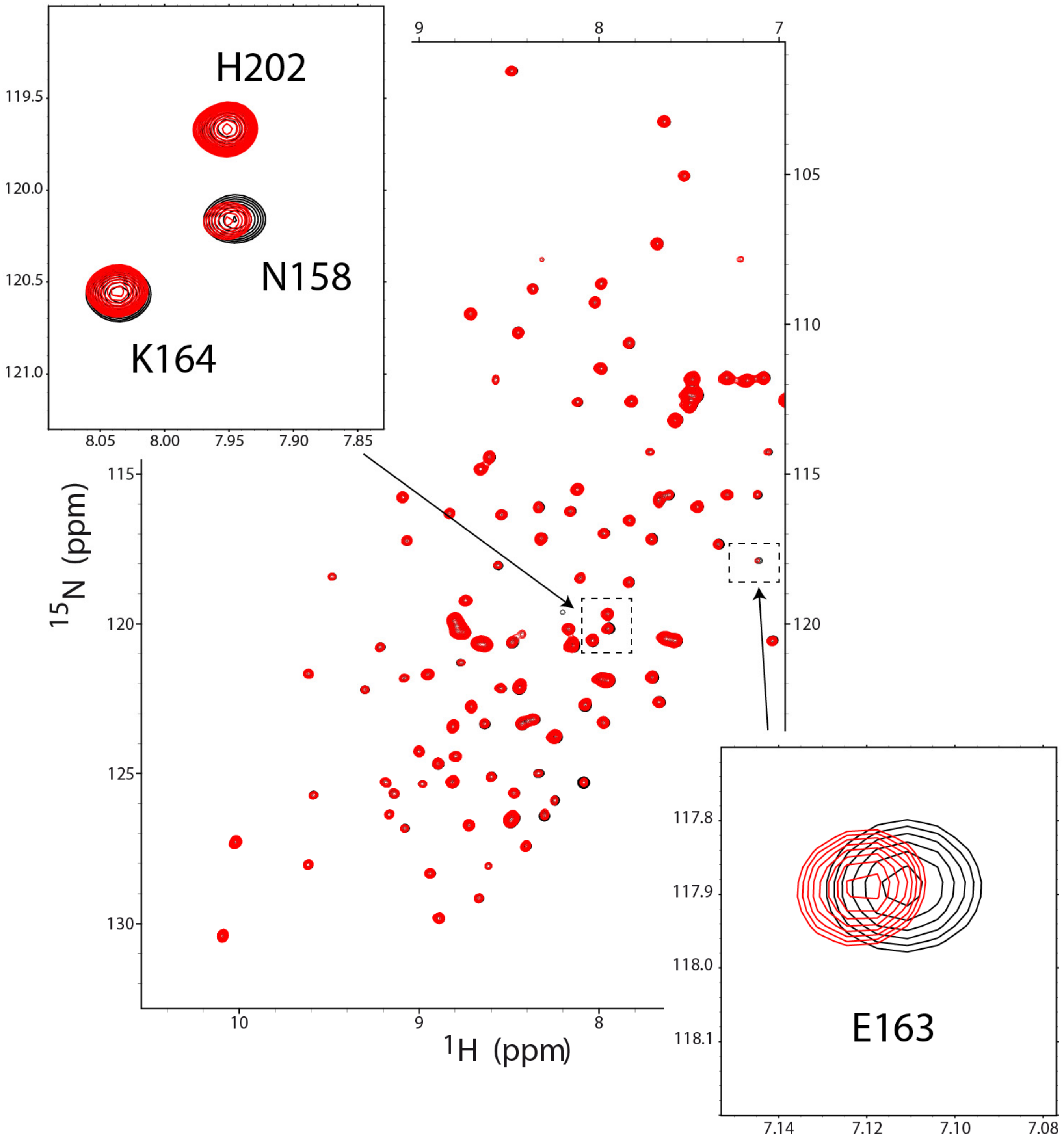

Figure S6. Superimposed ^{1}H-^{15}N HSQC spectra of free 0.2 mM ^{15}N-D2 (black), D2:DOBE in stoichiometric ratios 1:4 (red). Representative spectral regions are zoomed.

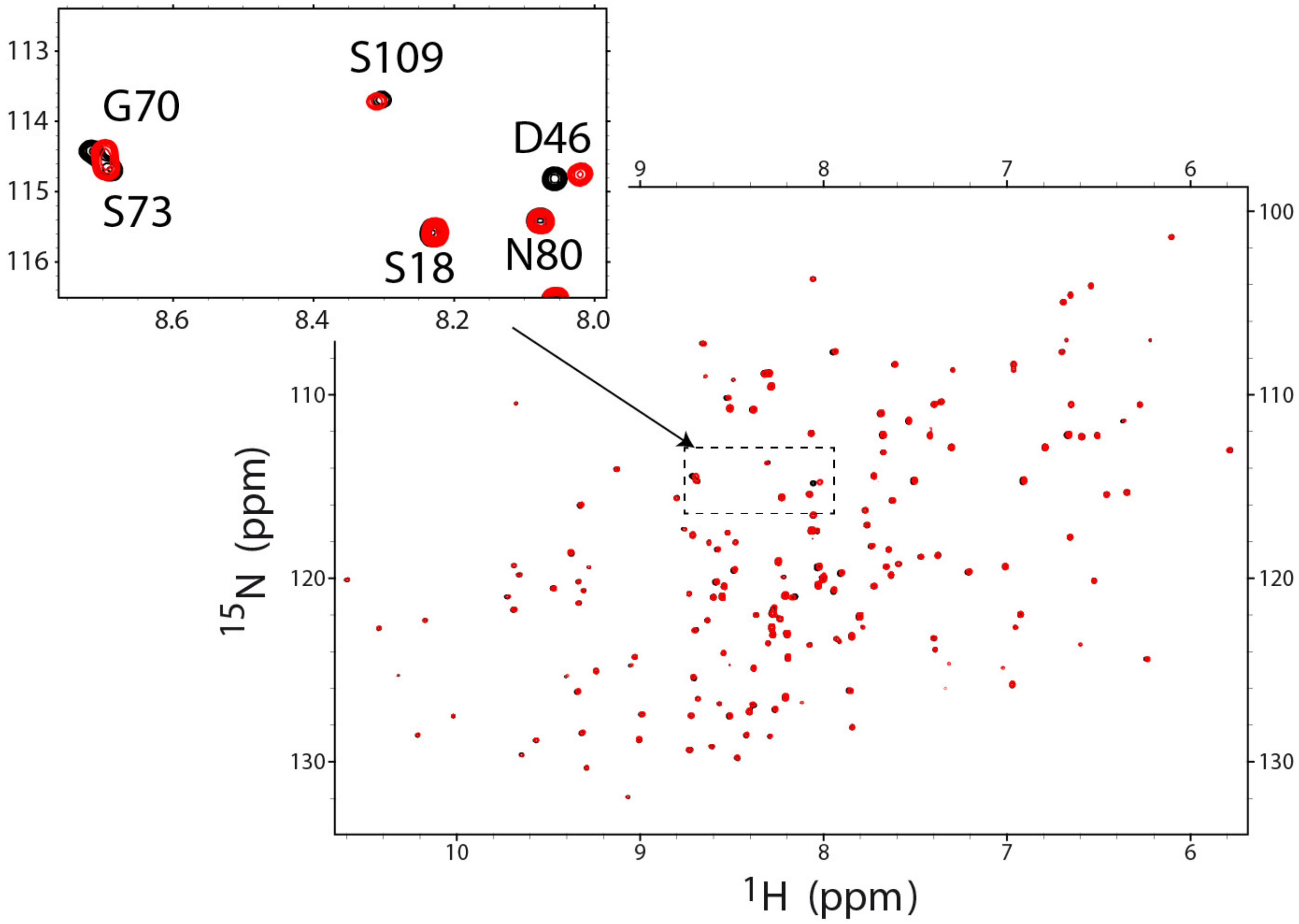

Figure S7. Superimposed ^{1}H-^{15}N HSQC spectra of free 0.2 mM ^{15}N-FGF2 (black), FGF2:RA in stoichiometric ratios 1:4 (red). A representative spectral region is zoomed.

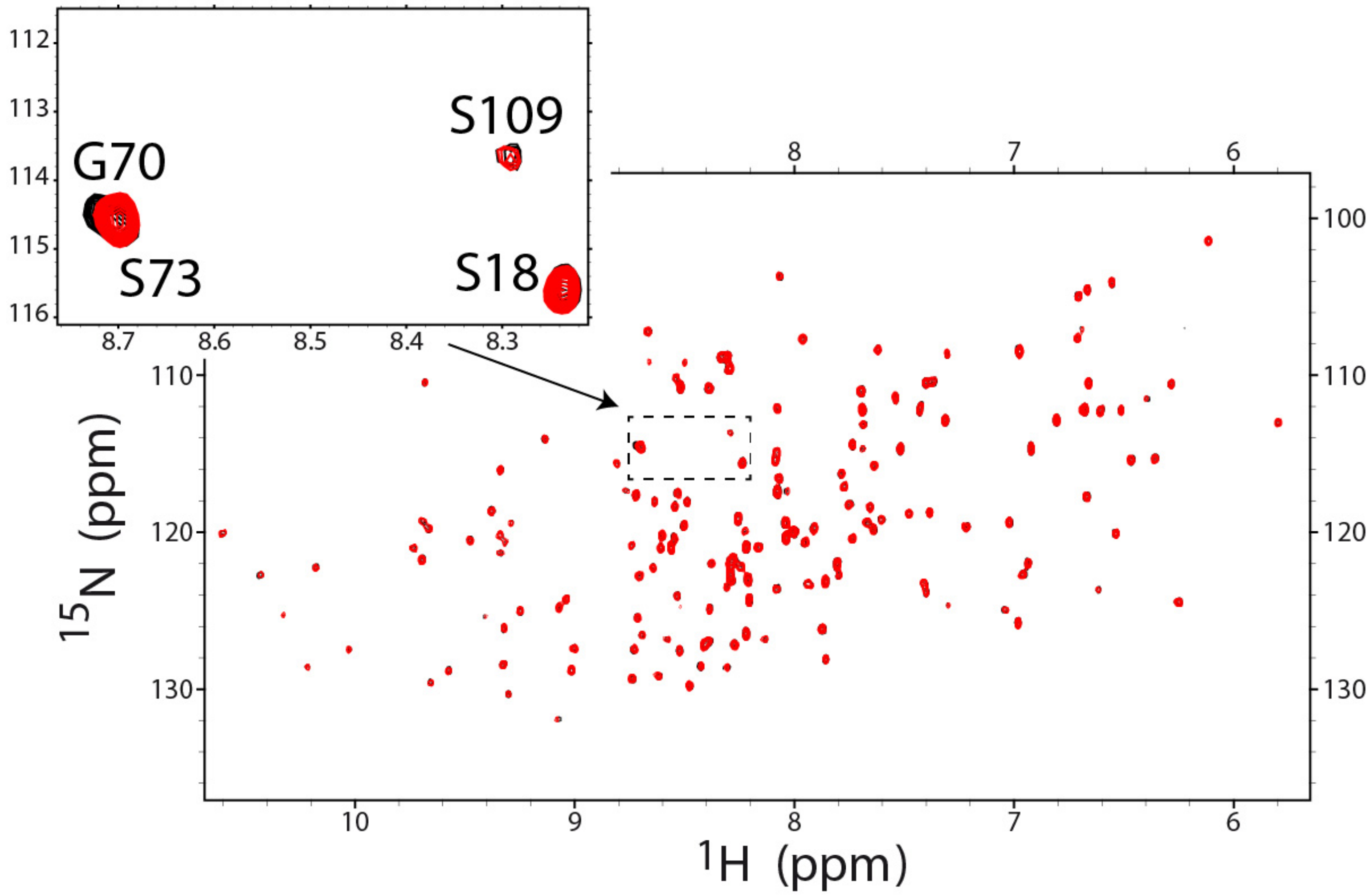

Figure S8. Superimposed ^{1}H-^{15}N HSQC spectra of free 0.2 mM ^{15}N-FGF2 (black), FGF2:RES in stoichiometric ratios 1:4 (red). A representative spectral region is zoomed.

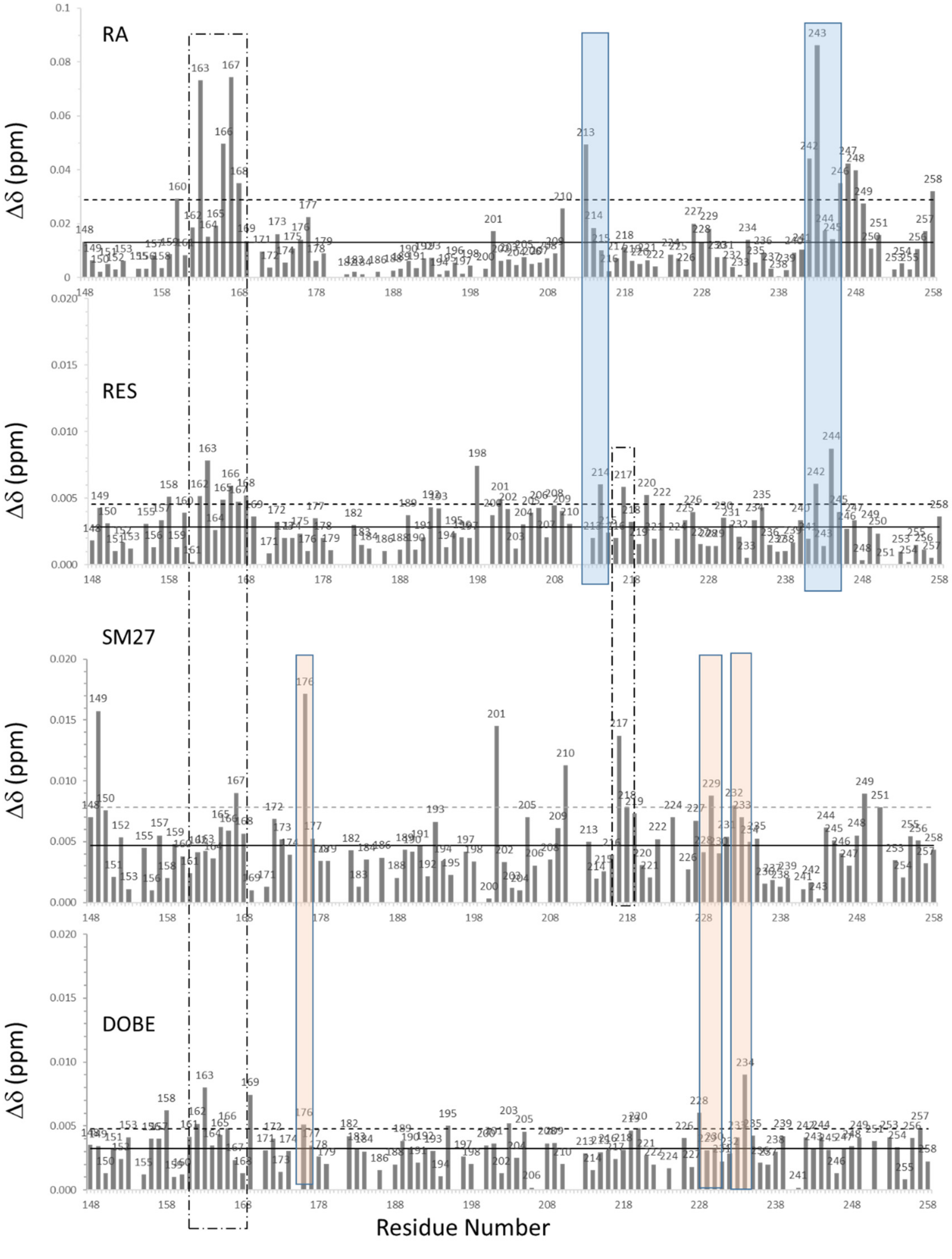

Figure S9. CSP effects on ^{15}N-D2 domain upon RA, RES, SM27 and DOBE addition observed for ^{15}N-D2:ligand 1:4 samples. Continuous and dashed lines indicate the CSP average values (⟨CSP⟩), and ⟨CSP⟩ + 1σ, respectively. Dashed and colored boxes highlight regions of common residues affected by resonance shifts upon ligand addition.

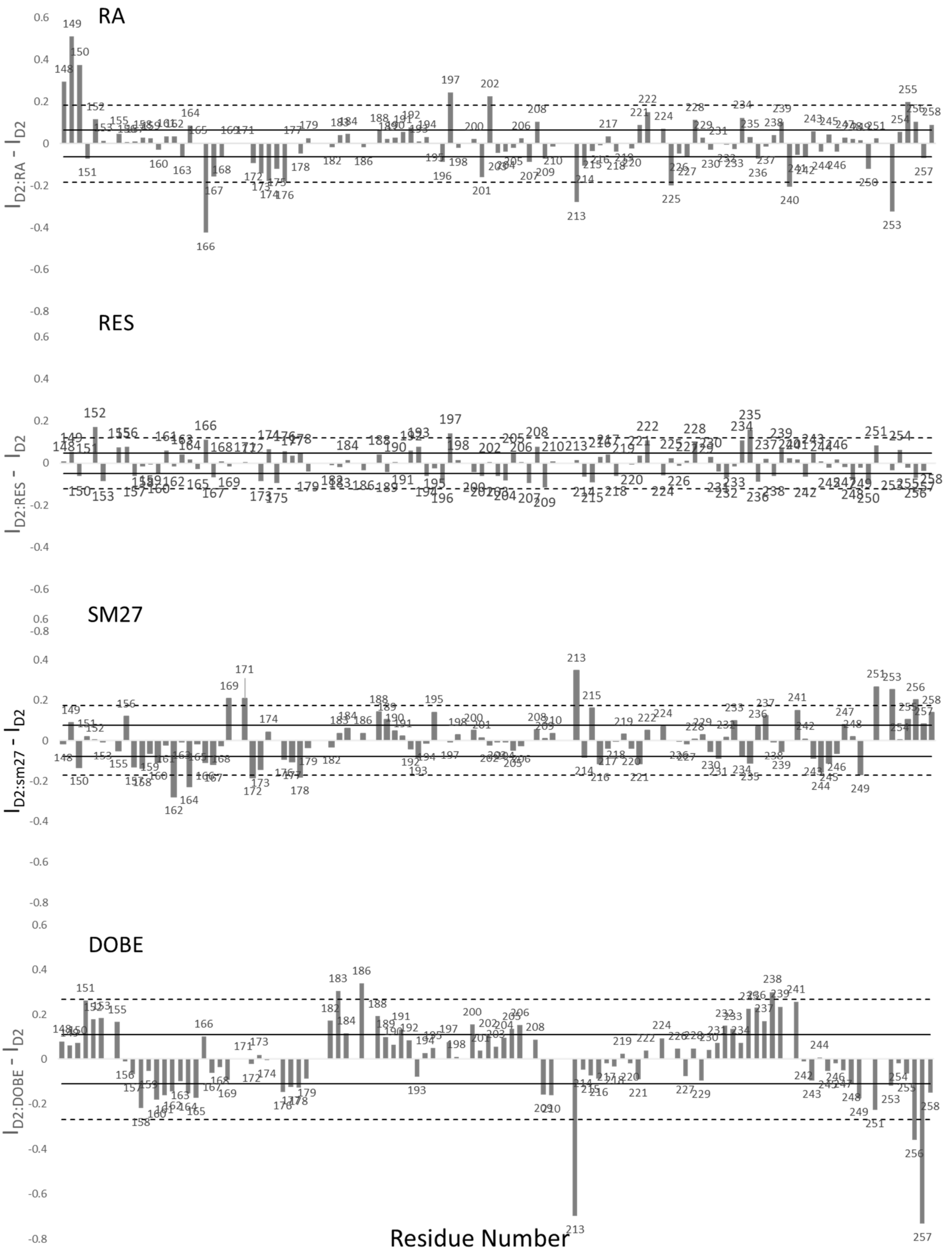

Figure S10. D2 normalized intensities differences in the presence and in the absence of RA, RES, SM27 and DOBE ligands. Straight and dashed lines represent $\pm \langle|I_{D2:ligand}-I_{D2}|\rangle$ and $\pm (\langle|I_{D2:ligand}-I_{D2}|\rangle + 2\sigma)$, respectively

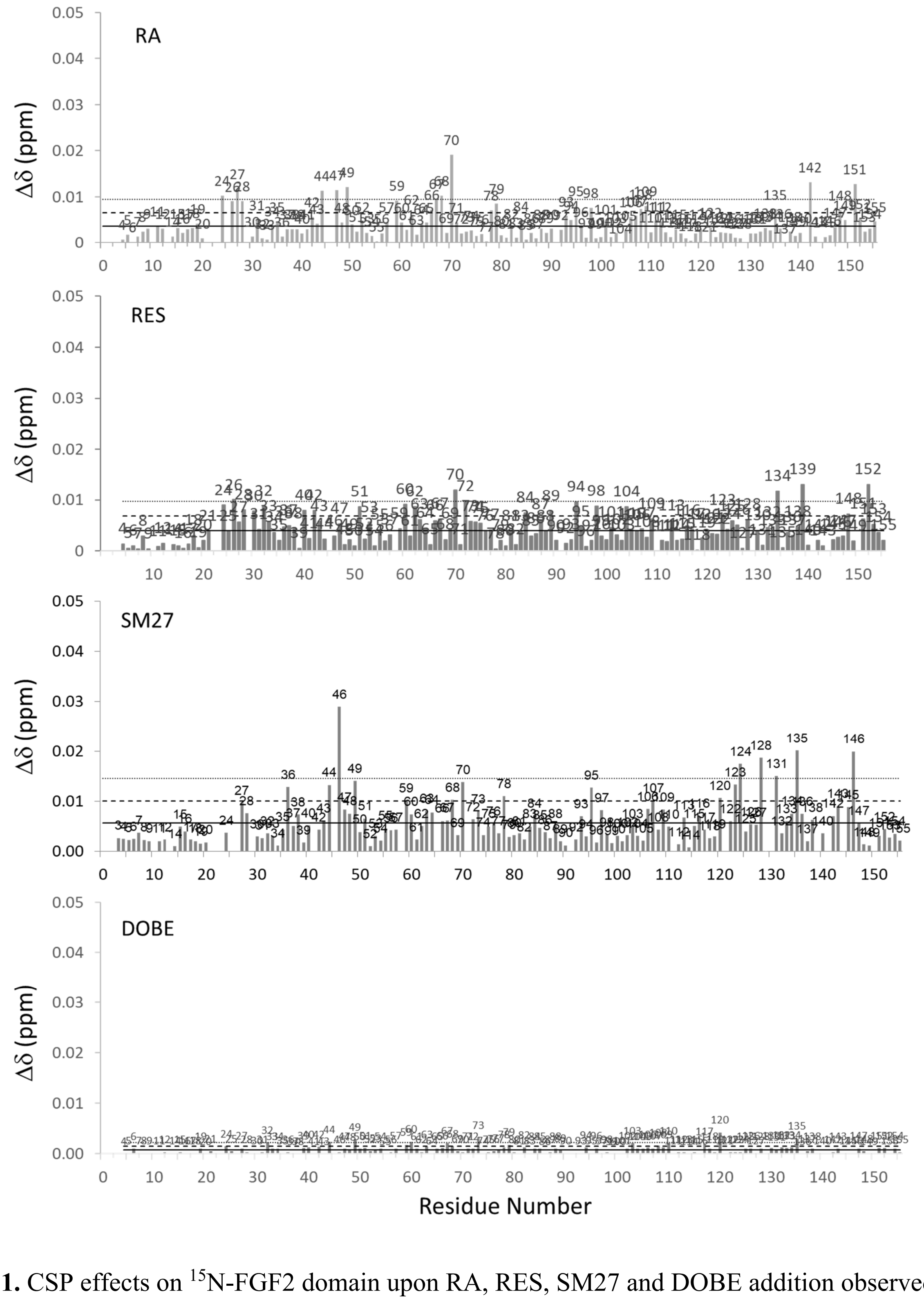

Figure S11. CSP effects on ^{15}N-FGF2 domain upon RA, RES, SM27 and DOBE addition observed for ^{15}N-D2:ligand 1:4 samples. Continuous, dashed, and dotted lines indicate the CSP average values (⟨CSP⟩), ⟨CSP⟩ + 1σ, and ⟨CSP⟩ + 2σ, respectively.

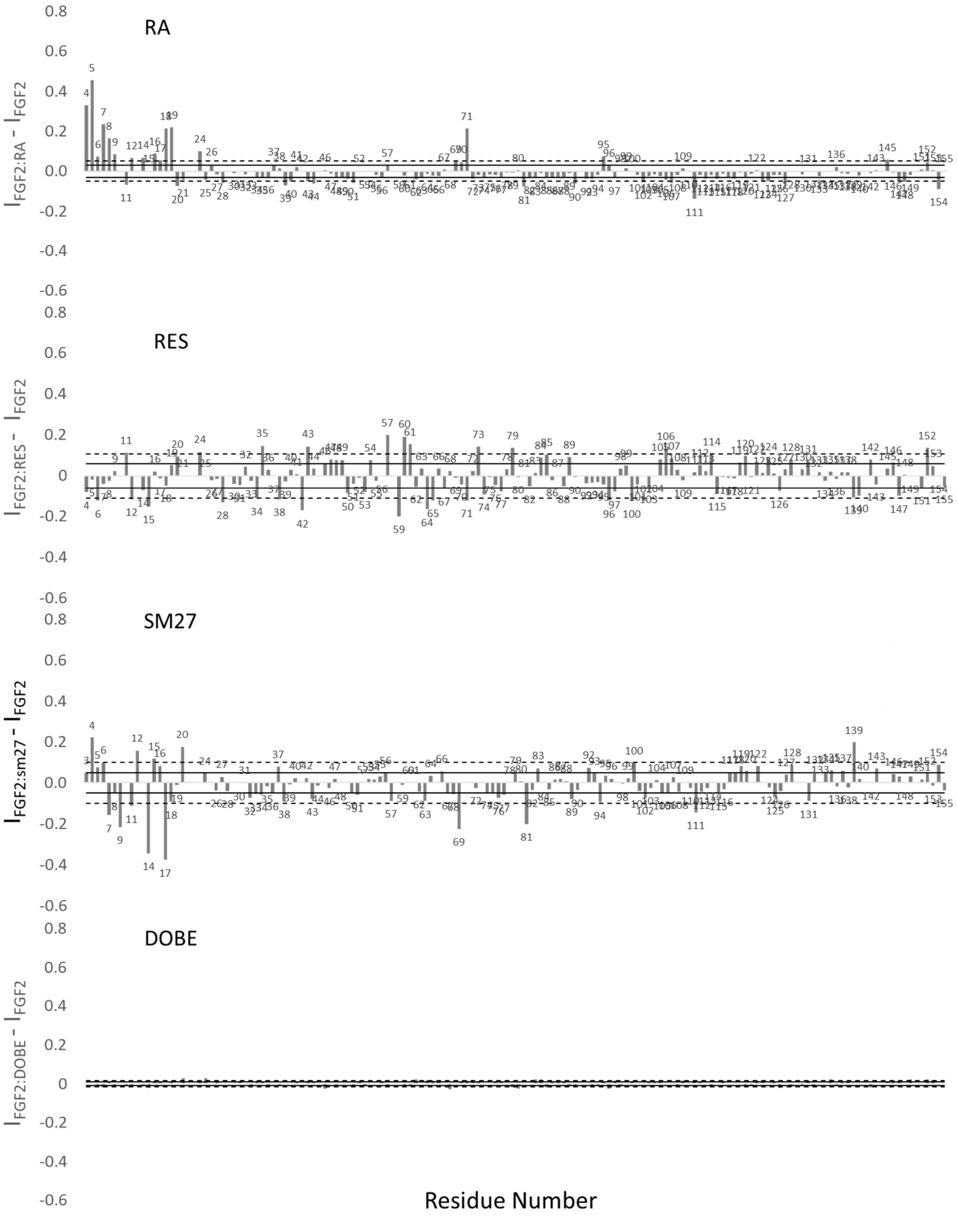

Figure S12. FGF2 normalized intensities differences in the presence and in the absence of RA, RES, SM27 and DOBE ligands. Straight and dashed lines represent $\pm \langle|I_{FGF2:ligand}\text{-IFGF}_2|\rangle$ and $\pm (\langle|I_{FGF2:ligand}\text{-}I_{FGF2}|\rangle + 2\sigma)$, respectively.

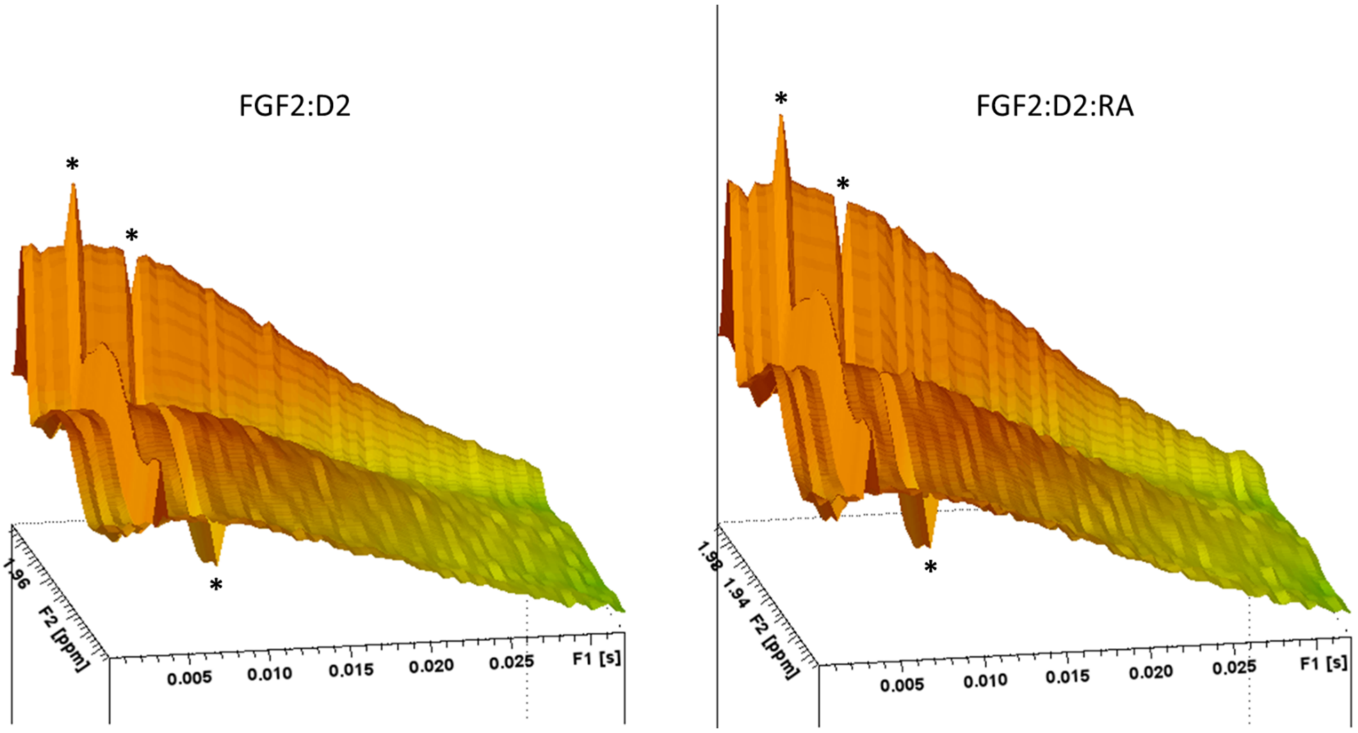

Figure S13 Selected diffusion profiles observed for aliphatic region of DOSY spectra recorded for FGF2:D2 complex in the absence (left) and presence (right) of RA. D values estimated from data fitting are D = 1.11 ± 0.02 (left panel, FGF2:D2) and D = 1.19 ± 0.02 (right panel, FGF2:D2:RA). Spikes labelled with * are due to residual water artefacts.
